# High Throughput Screens Yield Small Molecule Inhibitors of *Leishmania* CRK3:CYC6 Cyclin-Dependent Kinase

**DOI:** 10.1371/journal.pntd.0001033

**Published:** 2011-04-05

**Authors:** Roderick G. Walker, Graeme Thomson, Kirk Malone, Matthew W. Nowicki, Elaine Brown, David G. Blake, Nicholas J. Turner, Malcolm D. Walkinshaw, Karen M. Grant, Jeremy C. Mottram

**Affiliations:** 1 Wellcome Trust Centre for Molecular Parasitology, Institute of Infection, Immunity and Inflammation, College of Medical, Veterinary and Life Sciences, University of Glasgow, Glasgow, United Kingdom; 2 Cyclacel Ltd., Dundee, Dundee, United Kingdom; 3 Manchester Interdisciplinary Biocentre, University of Manchester, Manchester, United Kingdom; 4 Institute of Structural and Molecular Biology, The University of Edinburgh, Edinburgh, United Kingdom; 5 School of Health & Medicine, Division of Medicine, Lancaster University, Lancaster, United Kingdom; University of Tokyo, Japan

## Abstract

**Background:**

*Leishmania* species are parasitic protozoa that have a tightly controlled cell cycle, regulated by cyclin-dependent kinases (CDKs). Cdc2-related kinase 3 (CRK3), an essential CDK in *Leishmania* and functional orthologue of human CDK1, can form an active protein kinase complex with *Leishmania* cyclins CYCA and CYC6. Here we describe the identification and synthesis of specific small molecule inhibitors of bacterially expressed *Leishmania* CRK3:CYC6 using a high throughput screening assay and iterative chemistry. We also describe the biological activity of the molecules against *Leishmania* parasites.

**Methodology/Principal Findings:**

In order to obtain an active *Leishmania* CRK3:CYC6 protein kinase complex, we developed a co-expression and co-purification system for *Leishmania* CRK3 and CYC6 proteins. This active enzyme was used in a high throughput screening (HTS) platform, utilising an IMAP fluorescence polarisation assay. We carried out two chemical library screens and identified specific inhibitors of CRK3:CYC6 that were inactive against the human cyclin-dependent kinase CDK2:CycA. Subsequently, the best inhibitors were tested against 11 other mammalian protein kinases. Twelve of the most potent hits had an azapurine core with structure activity relationship (SAR) analysis identifying the functional groups on the 2 and 9 positions as essential for CRK3:CYC6 inhibition and specificity against CDK2:CycA. Iterative chemistry allowed synthesis of a number of azapurine derivatives with one, compound 17, demonstrating anti-parasitic activity against both promastigote and amastigote forms of *L. major*. Following the second HTS, 11 compounds with a thiazole core (active towards CRK3:CYC6 and inactive against CDK2:CycA) were tested. Ten of these hits demonstrated anti-parasitic activity against promastigote *L. major*.

**Conclusions/Significance:**

The pharmacophores identified from the high throughput screens, and the derivatives synthesised, selectively target the parasite enzyme and represent compounds for future hit-to-lead synthesis programs to develop therapeutics against *Leishmania* species. Challenges remain in identifying specific CDK inhibitors with both target selectivity and potency against the parasite.

## Author Summary

CRK3, a cdc2-related serine/threonine protein kinase of the CDK family, is essential for transition through the G2-M phase checkpoint of the *Leishmania* cell cycle. An expression and purification system has been developed to produce active *L. major* CRK3 in complex with a cyclin partner, CYC6. CRK3:CYC6 was used to develop an assay suitable for high throughput screening (HTS) using IMAP fluorescence polarization technology. Two compound chemical libraries were screened against CRK3:CYC6 and counter screened against a human cyclin-dependent kinase complex CDK2:CycA. Two main chemical families of inhibitors were identified that specifically inhibited the leishmanial cyclin-dependent kinase, the azapurines and the thiazoles. Structure activity relationship (SAR) analysis of the hits identified the chemical groups attached to the azapurine scaffold that are essential for the inhibition of CRK3:CYC6 protein kinase activity. The CRK3:CYC6 hits were subsequently tested against a panel of 11 mammalian kinases including human CDK1:CYCB, human CDK2:CYCA and human CDK4:CYCD1 to determine their selectivity. Compounds selective to CRK3:CYC6 were tested against *Leishmania*. Progress towards synthesising potent and selective derivatives of the HTS hits are discussed, with the view to evaluating their potential for the development of novel therapeutics against leishmaniasis.

## Introduction

The leishmaniases are a group of diseases caused by *Leishmania*, parasitic protozoa belonging to the family Trypanosomatidae. There are over 20 known species and sub species of *Leishmania* prevalent in 88 countries worldwide. These can be grouped into old world (Africa, Asia and Europe) and new world (the Americas) species according to their geographic distribution. (www.who.int/leishmaniasis/burden/en/). Several clinical forms of the disease occur; localised cutaneous, diffuse cutaneous, mucocutaneous, and visceral leishmaniasis. An estimated 350 million people are at risk of infection [1] with an estimated 12 million individuals infected worldwide. There is an annual incidence of 0.5 million of the visceral form of the disease and 1.5–2 million cases of the cutaneous form of the disease [2].

There are a number of drugs currently recommended for the treatment of leishmaniasis such as the pentavalent antimonials, Sodium stibogluconate (Pentostam, SSG) and Meglumine antimoniate (Glucantime); Amphotericin B and its lipid formulation AmBisome; Pentamidine, Miltefosine (Impavido) and Paromomycin [3]. Two more drugs (Imiquimod and Sitamaquine) are currently being assessed in clinical trials. However, the current repertoire of drugs for leishmaniasis is inadequate for a variety of reasons; high toxicity, poor efficacy, high cost, undesirable route of administration, narrow therapeutic window and drug resistance. Indeed extensive drug resistance to the pentavalent antimonials, has been reported in India [3]. Therefore there is an urgent need to develop new therapeutics to treat leishmaniasis and one area under investigation is the cell cycle and protein kinases [4], [5].

A number of diseases are attributed to defects in protein kinase-controlled cell signalling pathways, including cancer and inflammatory disease [6], [7], opening up the possibility of designing protein kinase inhibitors to rectify these defects. Indeed, Imatinib (Gleevec), which inhibits the Ableson tyrosine kinase (Abl), is already licensed to treat Chronic Myeloid Leukaemia (CML) [8]. Several small chemical inhibitors of cyclin-dependent kinases (CDKs) are undergoing clinical trial to assess their effectiveness in treating cancer. The rationale for their development stems from the fact that dysregulation of CDK signalling in many cancers results in unchecked proliferation [9]. Notable examples include alvocidib (Flavopiridol) and seliciclib (CYC202 or *R*-roscovitine). Alvocidib was the first CDK inhibitor to reach clinical trials [10]; it is a non-purine CDK inhibitor that inhibits a broad range of CDKs and other intracellular targets [11], [12]. It can induce cell cycle arrest at both the G1-S and G2-M boundaries [13] and inhibits the growth of a number of solid tumor cell lines [14]. Seliciclib is a more selective CDK inhibitor and has demonstrated antitumour activity against human tumour xenografts [15].

Studies on the yeast and mammalian cell cycles have established the key CDKs and cyclins that are involved in cell cycle regulation. This work is relevant to the study of the parasite cell cycle since homologues of many of these cell cycle regulatory proteins have been identified in protozoan parasites, for example: CRK3 in *Leishmania* [16] and *T. brucei* [17]; mitotic cyclins in *Trypanosoma brucei* [18]. Due to their pivotal role in the cell cycle, these proteins offer an attractive area for drug discovery and development against trypanosomatids.

Analysis of the genome from the three trypanosomatid protozoan parasites, *L. major*, *T. brucei* and *T. cruzi*, reveals that the CDK family in trypanosomatids is relatively large, compared with other unicellular organisms, with 11 in *T. brucei* and *L. major* and 10 in *T. cruzi*. Moreover, 10 putative cyclins, CYC2-11, have been identified in all three parasites [4]. *Leishmania* possess an additional cyclin, CYCA, which is absent from both *T. brucei* and *T. cruzi*.

As anticipated, evidence suggests that trypanosomatid CDKs control the parasite cell cycle and that interaction with cyclins is crucial to this activity. The *L. major* CDK, CRK3, can complement a temperature sensitive *S. pombe* cdc2 null mutant [19], demonstrating its functional homology to cdc2/CDK1. The gene for *L. mexicana* CRK3 (99% identical to *L. major* CRK3) is essential, as befits a crucial regulator of cell division. CRK3 activity was found to peak in the G2/M phase of the cell cycle and inhibition of CRK3 *in vivo* resulted in cell cycle arrest [20]. Sequence analysis indicates that CRK3 contains residues and domains conserved in other organisms; PSTAIRE domain, involved in cyclin binding; Thr-14 and Tyr-15, which are required for ATP binding, and Thr-161, the T-loop residue, phosphorylated by a CDK activating kinase [21]. In the current study we reconstituted active CRK3:CYC6 complex *in vitro*; determined the optimal peptide substrate for the complex; adapted a high-throughput robotic assay for use with CRK3:CYC6; screened approximately 30,000 compounds and discovered new parasite-selective pharmacophores that could be developed into therapeutics to treat the leishmaniases and shorten the drug discovery process.

## Materials and Methods

### *Leishmania* CRK3:CYC6 protein kinase complex co-expression and co-purification

*E. coli* BL21 (DE3) pLys-S cells were transformed with plasmid pGL1218 (CYC6his) and plated on an LB-agar plate with ampicillin (50 µg ml^−1^) and chloramphenicol (38 µg ml^−1^) antibiotics. CYC6-expressing bacteria were then re-transformed with plasmid pGL751a (CRK3his) and plated onto an LB-agar plate supplemented with kanamycin (25 µg ml^−1^), ampicillin (50 µg ml^−1^) and chloramphenicol (38 µg ml^−1^) antibiotics. A single colony of co-transformed *E. coli* BL21 (DE3) pLys-S cells were used to inoculate 5 ml of LB-medium with kanamycin (25 µg ml^−1^), ampicillin (50 µg ml^−1^) and chloramphenicol (38 µg ml^−1^) antibiotics and grown with agitation at 37°C overnight. The 5 ml bacterial culture was diluted to l litre with LB medium plus antibiotics and the culture grown at 37°C until it reached anOD_600 nm_ of 0.7. The 1 litre culture was then shifted to the induction temperature of 19°C for 30 minutes and protein expression induced with 1 mM IPTG. Cultures were induced at 19°C over night with agitation. After 16 hours, cells were harvested at 4000× g for 15 minutes and resuspended in ice-cold PBS pH 7.4 supplemented with DNAse-1 (10 µg ml^−1^) (Invitrogen) and Lysozyme (100 µg ml^−1^) (Sigma) for 60 minutes on ice. The cell lysate was sonicated 4×30 sec (30 sec. on/30 sec. off), harvested at 12000× g for 20 minutes and the soluble extract filtered through a 0.2 µm filter syringe. The proteins were purified via BioCAD chromatography using a metal chelate Ni^2+^ charged column followed by a Hiload 16/60 Superdex-200 gel filtration column. The bacterial cell lysate was loaded onto the Ni^2+^ column pre-equilibrated with wash buffer (50 mM Na_2_HPO_4_, 300 mM NaCl pH 8.0 and 50 mM imidazole) and non-specifically bound proteins removed by washing with the Ni^2+^ column wash buffer. CRK3:CYC6 was eluted at 1 ml min^−1^ with a linear gradient of 50–500 mM imidazole in wash buffer, over 10 column volumes (1 column volume = 1.75 ml). The fractions containing the most protein, detected by absorbance at 280 nm, were pooled and loaded onto a Hiload 16/60 Superdex-200 gel filtration column pre-equilibrated with gel filtration buffer/enzyme storage buffer (20 mM HEPES pH 7.4, 50 mM NaCl, 2 mM EGTA, 2 mM DTT and 0.02% Brij-35). The complex was eluted at 1 ml min^−1^ with gel filtration buffer/enzyme storage buffer and the fractions collected. The fractions containing CRK3his and CYC6his proteins were determined both by Coomassie blue gel staining and Western blot analysis and subsequently pooled together. The pooled fractions had glycerol added to 10% of the final volume along with Roche EDTA-free complete protease inhibitors, were aliquoted and stored at −80°C.

### Expression and purification of individual proteins

*Escherichia coli* BL21 (DE3) pLys-S strains were transformed with either CRK3 plasmid DNA (pGL751a) or CYC6 plasmid DNA (pGL1218). Transformed cells were plated onto an LB-agar plate supplemented with kanamycin (25 µg ml^−1^)/chloramphenicol (38 µg ml^−1^) for CRK3 and ampicillin (50 µg ml^−1^)/chloramphenicol (38 µg ml^−1^) for CYC6. A single colony was inoculated into 5 ml of LB-media with the appropriate antibiotics and grown with agitation overnight at 37°C. Bacterial cultures were bulked up to an appropriate volume and grown at 37°C in LB-media supplemented with the appropriate antibiotics to an optical density of 0.7 at a wavelength of 600 nm (O.D._600 nm_). Cultures were shifted to their 19°C induction temperature for 30 mins before protein expression was induced over night using Isopropyl-β-D-Thiogalactopyranoside (IPTG) (300 µM for CRK3 and 1 mM for CYC6) at 19°C. Cells were harvested at 4000× g for 15 minutes and resuspended in ice-cold PBS, pH 7.4 supplemented with DNAse-I (10 µg ml^−1^) and Lysozyme (100 µg ml^−1^) and incubated for 60 minutes on ice. The cell lysate was sonicated 5×15 seconds (1 sec. on/1 sec. off) to break open the cells and harvested at 12000× g for 20 minutes. The proteins were purified via BioCAD chromatography using a metal chelate Ni^2+^ charged column. Proteins were loaded onto the Ni^2+^ column and the flow through collected. The column was washed with Ni^2+^ column loading/wash buffer (50 mM Na_2_H_2_PO_4_, 300 mM NaCl pH8.0 and 50 mM imidazole) to remove non-specific proteins bound to the column, and the wash collected. Proteins were eluted using a Ni^2+^ column elution buffer (50 mM Na_2_H_2_PO_4_, 300 mM NaCl pH8.0) with a gradient of 50–500 mM imidazole over 10 column volumes (1 column volume = 1.75 ml). For *L. major* CYC6 only, Ni^2+^ purification was followed by purification on a strong anion exchange Poros HQ 10 micron 4.6 mmD/100 mmL column (Applied Biosystems). The CYC6-containing fractions were pooled and passed through a PD-10 desalting column (Amersham) before being loaded onto the strong anion exchange column. Proteins were eluted using the anion exchange column elution buffer (50 mM Tris, 5 mM EDTA pH7.0 and a 0–1 M NaCl gradient) and the fractions collected. The identity of purified proteins were confirmed by mass spectrometry.

### Protein kinase assays

#### γ-^32^P gel-based assays

Protein kinase assays were performed using CRK3 and CYC6 cell cycle proteins in a final volume of 20 µl. Assays were performed using the kinase assay buffer (KAB) (50 mM MOPS, pH 7.2, 20 mM MgCl_2_, 10 mM EGTA and 2 mM DTT) supplemented with 4 µM ATP, 0.5 µCi of 3000 Ci/mmole γ-^32^P ATP (Perkin-Elmer) per reaction and histone H1 as a substrate used at 0.25 mg ml^−1^. Assays were carried out for 30 minutes at 30°C before stopping the reaction by the addition of 7.5 µl of 4× SDS-PAGE sample buffer. The samples were incubated at 100°C for 5 minutes and electrophoresed on a 12% SDS-PAGE gel. Gels were processed by staining with Coomassie blue R250 for 20 minutes, rinsing with distilled water and destaining to remove the excess Coomassie stain. Gels were then dried before overnight exposure to KODAK autoradiography film for 16 hours, and developed by a Kodak X-omat automated developer.

#### γ-^32^P microtiter radiometric assays

*Leishmania* CRK3:CYC6 protein kinase assays were performed in 96-well microtiter plates in a final volume of 25 µl. Each assay point contained 7.5 ng of co-expressed histidine-tagged CRK3:CYC6 protein kinase complex (hereafter referred to as CRK3:CYC6) diluted in enzyme dilution buffer (EDB) (20 mM Tris-HCl pH 7.2, 0.5 mg ml^−1^ BSA, 2.5% glycerol and 0.006% Brij-35). Assays were performed using the assay development buffer (ADB) (20 mM MOPS, pH 7.0, 25 mM β-glycerophosphate, 5 mM EGTA, 1 mM NaVO_3_, 1 mM DTT and 15 mM MgCl_2_) supplemented with 100 µM ATP, 0.5 µCi of γ-^32^P ATP per reaction and histone H1 as a substrate used at 0.4 mg ml^−1^. Two and three-fold titrations were set up to determine the concentration of enzyme to be used per assay point (7.5 ng) and to determine the concentrations of selected inhibitors required for 50% inhibition of *Leishmania* CRK3:CYC6 protein kinase activity (IC_50_ values), respectively. For IC_50_ determinations, assay mixes contained DMSO to a final concentration of 2%. Mammalian protein kinase assays were carried out according to the assay protocols developed at Cyclacel. Assays were carried out for 30 minutes at 30°C before stopping the reaction by the addition of an equal volume (25 µl) of 75 mM orthophosphoric acid. Samples were spotted onto a p81 cellulose filterplate (Nunc) and a vacuum applied. Wells were washed 3×200 µl with 75 mM phosphoric acid and the bottom of the plate sealed. 50 µl of Microscint 40 (Perkin Elmer) was added per well before incorporation of radioactivity was determined on a Topcount microplate scintillation counter.

#### IMAP fluorescence polarization assays

Protein kinase assays were performed in 384-well non-treated black plates (Nunc) in a final volume of 20 µl. Each assay point contained 1.25 ng of co-expressed *Leishmania* CRK3:CYC6 protein kinase complex. Assays were performed using enzyme complex, 100 nM fluorescently labelled peptide substrate (5FAM-GGGRSPGRRRRK-OH) (Molecular Devices), 100 µM ATP and plus or minus an inhibitor. The enzyme complex, peptide and ATP were made up in the IMAP complete reaction buffer (CRB) (10 mM Tris-HCl, pH 7.2, 10 mM MgCl_2_, 0.05% NaN_3_, 0.01% Tween-20 and 1 mM DTT). Assays were carried out for 1 hour 20 minutes at room temperature and the reaction stopped by the addition of 50 µl of the IMAP progressive binding reagent (Proprietary buffer from Molecular Devices plus tri-valent metal-containing nanoparticles). The assay was left to proceed for a further 1 hour 20 minutes at room temperature and the fluorescence polarization determined by a Perkin Elmer Fusion microplate reader, with excitation at 485 nm and emission at 535 nm.

### Compound libraries

A compound library provided by Cyclacel under its license from Lexicon Pharmaceuticals Inc. contained approximately 25,000 compounds composed of two sub libraries, the heterocycle 2 (HL-2) and kinase inhibitor theme libraries. The HL-2 sub library contained approximately 16,000 compounds and included 6 synthetic themes and 10 heterocyclic themes. These compounds were designed to include desirable pharmaceutical properties such as following Lipinski rules [22] and ADME (absorption, distribution, metabolism and excretion) properties [23]. The kinase inhibitor theme library contained approximately 8,000 and included heterocycle 1 (HL-1) compounds in addition to published adenine, pyrimidine, quinazoline and quinoxaline kinase inhibitors. It also comprised of natural product mimicking compounds [24] such as sugar nucleoside mimics, protease inhibitor themes, steroid mimics, aminoglycoside mimics and phosphatase inhibitor themes. The synthesis of novel azapurine ligands is described in supporting information (see S3 in Text S1).

The BioFocus compound library contained 4596 compounds and comprised of a kinase and ThemePair library (Galapagos N.V.; www.glpg.com). The kinase library was further divided into seven sub libraries including DFG out, hinge binding and novel binding compounds (www.biofocus.com) [21]. The ThemePair library contained 20 different compound scaffolds which were fragment-like and highly soluble. Of these, the most promising sub library was the SFK-48 kinase focused library (Galapagos N.V.) and this was chosen for further testing against *Leishmania* CRK3:CYC6. These compounds contained variable groups which were designed *in silico* by BioFocus to explore the ‘DFG out’ conformation.

### Molecular modelling

A model of the active cyclin-bound structure of CRK3 was built by by aligning the sequences of LmajCRK3 (residues 1–311, accession code O96526) with human CDK2. The alignment was then used to build a model of the complex using the comparative protein structure modelling program, MODELLER [25]. The crystal structure of the human CDK2-CYCA complexed with the small molecule inhibitor indirubin-5-sulfonate (pdb code 1E9H) was used as the model template. Of the 20 recorded solutions, the model with the lowest energy was used as the final model. 3D structures of the small molecule inhibitors were built using PRODRG [26]. Manual docking was carried out using the program Pymol.

### Parasite culture

*L. major* wild type promastigotes (Friedlin strain: WHO designation MHOM/JL/81/Friedlin) were grown at 25°C in HOMEM medium (Invitrogen cat no. 041-94699111) supplemented with 10% (v/v) heat inactivated foetal calf serum (HIFCS) and 1% (v/v) penicillin/streptomycin antibiotics.

### *L. major* promastigote growth inhibition assay

CRK3 inhibitors were diluted into HOMEM medium supplemented with 10% HIFCS at twice the final screening concentration. Five-fold or ten-fold serial dilutions were carried out into HOMEM medium supplemented with 10% HIFCS. 100 µl of each drug concentration were added to a 96 well plate in duplicate. 100 µl of five-fold or ten-fold serial dilutions of 1 mM Pentamidine (Sigma) and an equivalent volume of 100% DMSO were included as positive and negative controls, in duplicate, respectively. *L. major* promastigote cells were diluted to a cell density of 2×10^6^ cells ml^−1^ in HOMEM media supplemented with 10% HIFCS and 100 µl added to all wells in 96 well plates. Plates were sealed with parafilm and incubated for 5 days at 25°C. After 5 days, 20 µl of filter sterile resazurin solution (12.5 mg resazurin salt in 100 ml PBS) (Sigma) was added to each of the wells and the plate incubated for a further 24 hours at 25°C. Fluorescence was measured using an Envision plate reader (Perkin Elmer) at 540 nm excitation wavelength and 590 nm emission wavelength [27].

### Macrophage (mΦ) extraction and purification

Macrophages were harvested from the peritoneum of Balb/C mice, centrifuged at 1000× g for 10 minutes at 4°C and resuspended in fresh RPMI 1640 media supplemented with 10% HIFCS and 1% (v/v) Gentamicin, as described previously [28]. Macrophages were diluted to a cell density of 5×10^5^ cells ml^−1^ in RPMI supplemented with 10% HIFCS and 100 µl were added to each well of a 16-well Lab-tek cavity slide (50,000 mφ/well) and incubated at 37°C, 5% CO_2_ for subsequent experiments. Macrophages were infected with *L. major* promastigotes at a ratio of 1∶8 (macrophage∶parasite) and the slides incubated for 24 hours at 37°C with 5% CO_2_. Inhibitors were set up in a five-fold dilution series in RPMI 1640 medium supplemented with 10% HIFCS. 200 µl of inhibitor were added to the wells in serial dilution and slides incubated for 72 hours at 37°C with 5% CO_2._ After 72 hours the medium was removed and replaced with fresh medium containing the same concentrations of inhibitors and incubated for a further 48 hours at 37°C with 5% CO_2_. At the end of the incubation period, the medium was removed, the slides were washed twice with fresh RPMI 1640 medium supplemented with 10% HIFCS then fixed with 100% methanol and stained with 10% Giemsa's stain for 10 minutes. The percentage of infected macrophages and number of amastigotes per macrophage were determined by light microscopy under oil immersion.

## Results

### Expression of active *Leishmania* CRK3:CYC6

**Figure 1:**
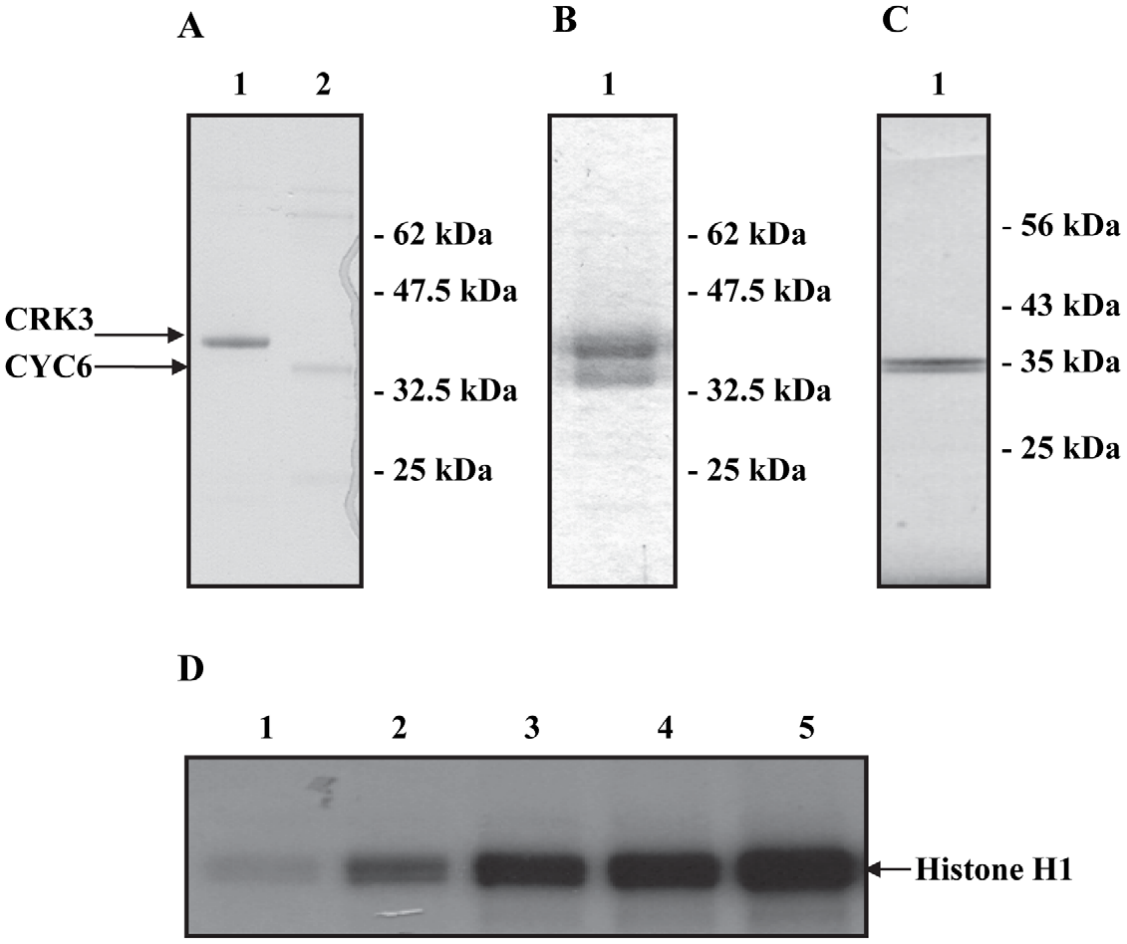
Expression and purification of the *Leishmania* CRK3:CYC6. (A) CRK3his and CYC6his were expressed in *E. coli* individually (lanes 1 and 2 respectively). Coomassie-stained SDS-PAGE. (B) Co-expressed CRK3his and CYC6his purified by Nickel-chelate and gel filtration chromatography. Coomassie-stained SDS-PAGE. (C) Co-expression of CRK3 and CYC6his and purification of the CRK3:CYC6his by Nickel-chelate and ion-exchange chromatography. Coomassie-stained SDS-PAGE. (D) Activation of CRK3his histone H1 kinase in the presence of increasing concentrations of CYC6his. All lanes contain 1.25 µg of CRK3 and 0.025 µg, 0.05 µg, 0.075 µg, 0.1 µg of CYC6 (lanes 2–5).

Three expression systems were devised to produce an active CRK3:CYC6 complex. Firstly, 38 kDa histidine-tagged CRK3 (CRK3his) and 35 kDa histidine tagged CYC6 (CYC6his) were expressed and purified from *E. coli* individually (Fig. 1A, lanes 1 and 2) and then combined to form a complex in a 1∶1 molar ratio. Secondly, CRK3his and CYC6his were co-expressed in *E. coli* and soluble protein was purified by nickel chelate and gel filtration chromatography (Fig. 1B). In this case the CRK3his was expressed at significantly higher levels than CYC6his, resulting in an excess of monomeric CRK3his. Monomeric CRK3 may be able to bind the inhibitors and thus alter their availability to bind and inhibit the active complex, so the gel filtration step was important to separate the CRK3:CYC6 complex from the free CRK3. This complex was used for screening (see below). To circumvent this problem and to provide active enzyme for detailed enzymatic analyses (data not shown), CYC6his was co-expressed with untagged CRK3 in *E. coli* and purified by Nickel chelate chromatography and ion exchange (Fig. 1C). This resulted in a homogenous preparation of CRK3:CYC6 complex with the subunits found in a 1∶1 molar ratio. The identities of the proteins were confirmed by peptide mass fingerprinting and the yield of the complex determined at ∼4.5 mg litre^−1^.

### Development of a protein kinase assay for CRK3:CYC6 suitable for HTS

*Leishmania* CRK3 is inactive when expressed and purified as a monomeric recombinant protein (Fig. 1D, lane 1), but is activated to produce a histone H1 kinase in the presence of either CYCA [29] or CYC6 (Fig. 1D, lanes 2–5). No auto-phosphorylation was detected, so the histone H1 kinase activity of CRK3:CYC6 is not dependent on phosphorylation of the T-loop threonine (residue T178 in *L. major* CRK3) [29], as has been reported for *S. cerevisiae* CDC28 [30]–[32] or human CDK1 [33]. A plate based radiometric protein kinase assay using histone H1 as a substrate was developed in order to test potential CRK3:CYC6 inhibitors. A ten-point, two-fold enzyme titration of CRK3:CYC6 was carried out and determined that 7.5 ng of protein complex produces a signal of approximately 15,000 cpm at the 30 min time point, in the linear phase of the assay, with a signal to background ratio of approximately 15∶1 (Figure S1 in Supporting Information Text S1). This was an acceptable starting point for further assay development and 7.5 ng of protein complex was used in all subsequent radiometric assays. The assay was validated with a Z′ score of 0.67, which is considered very good in terms of assay quality [34], [35].

The IMAP fluorescent polarisation assay was selected for the high throughput screen. First a substrate finder assay was carried out with 61 potential serine/threonine protein kinase substrates. This revealed that a generic sequence (GGGRSPGRRRRK) and two histone H1 derived peptides (GGGPATPKKAKKL and PKTPKKAKKL) gave the highest fluorescence polarization signals. Several other peptides were also found to have significant activity, including DYRKtide RRRFRPASPLRGPPK and a CDK7 derived peptide FLAKSFGSPNRAYKK. Analysis of the 5 peptide substrates highlighted that they all contained a sequence pattern xS/TPxR/K, which is in accordance with the optimal recognition motif for CDKs, x_−1_(S/T_0_)P_+1_x_+2_(K/R_+3_) [36] (Table 1). The generic peptide substrate was chosen as the optimum substrate and used in all subsequent IMAP assays. In order to establish the quantity of CRK3:CYC6 to use in the IMAP HTS assays, a two-fold enzyme titration was carried out (Figure S2 in Supporting Information Text S1). This identified that 1.25 ng of kinase complex could be used per assay point. When running the assay for 1 hour 20 minutes, this produced a signal of approximately 280 mP with a ΔmP of 180 mP, which was in the linear phase of the assay. The assay was validated under these conditions with a Z′ score of 0.71, showing it was reliable, robust and suitable for HTS [37].

**Table 1:** Analysis of the peptide substrates identified from the IMAP substrate finder assay.

Peptide	Sequence	Amino acid at −1 position	Amino acid at 0 position	Amino acid at +1 position	ΔmP signal
Generic Sequence	GGGRSPGRRRRK	Arginine	Serine	Proline	310
Histone H1 derived	GGGPATPKKAKKL	Alanine	Threonine	Proline	295
Histone H1 derived (aa 9–18)	PKTPKKAKKL	Lysine	Threonine	Proline	280
DYRKtide	RRRFRPASPLRGPPK	Alanine	Serine	Proline	190
CDK7 derived	FLAKSFGSPNRAYKK	Glycine	Serine	Proline	180

The five peptides identified as substrates for CRK3:CYC6 were analysed by sequence alignment. The consensus sequence pattern follows the optimal recognition motif identified for mammalian CDKs.

Sequence pattern: x S/T P x R/K.

Optimal recognition motif for CDKs: x−1 (S/T0) P+1 x+2 (K/R+3).

Underlined are the serine/threonine amino acid residues (in the 0 position) which are phosphorylated by the CRK3:CYC6 protein kinase complex.

### High throughput screens of *Leishmania* CRK3:CYC6

As cyclin-dependent kinases are amongst the most highly conserved protein kinases between human and *Leishmania*, we reasoned that selectivity should be built into the HTS screening protocol. *Leishmania* CRK3:CYC6 was screened against two compound libraries: firstly, the Lexicon library, which comprises a diverse set of 25,000 compounds, and secondly, the SFK48 kinase focused library from BioFocus. The first screen with the Lexicon library identified 43 compounds that produced a ≥50% inhibition of *Leishmania* CRK3:CYC6 protein kinase activity at 10 µM. As this library had already been screened against human CDK2:CycA, we were able to identify 43 compounds that inhibited the parasite enzyme, but not the human cyclin-dependent kinase (IC_50_>50 µM). Six of 43 hits were identified in follow up studies as false positive hits, whilst the remaining 37 were taken forward for IC_50_ determinations against CRK3:CYC6. 16 compounds had IC_50_ values ranging from 2.6–11 µM and 12 of those were azapurine compounds (Table 2).

**Table 2:** Lexicon azapurine HTS hits.

Compound	CRK3:CYC6IC_50_ (µM)	CDK4:CYCD1IC_50_ (µM)^a^	IC_50_ against WT promastigote *L. major* (µM)	IC_50_ against WT amastigote *L. major* (µM)
1	2.6	12.5	>10	>50
2	3.4	5.6	>10	38.4
3	4.4	21.9	>10	>50
4	4.4	10.3	>10	>50
5	5.3	9.7	8.6	>50
6	6.9	9.2	>10	>50
7	7.8	19.5	ND	ND
8	8.1	26.9	ND	ND
9	8.8	19.8	ND	ND
10	10.1	7.3	ND	ND
11	10.3	6.7	ND	ND
12	10.7	4.6	ND	ND

a Also screened against CDK1:CYCB and CDK2:CYCA; IC_50_ values for all compounds in these screens were >50 µM. ND, not determined (See Table S2 in Supporting Information Text S1 for compound structures.).

**Figure 2:**
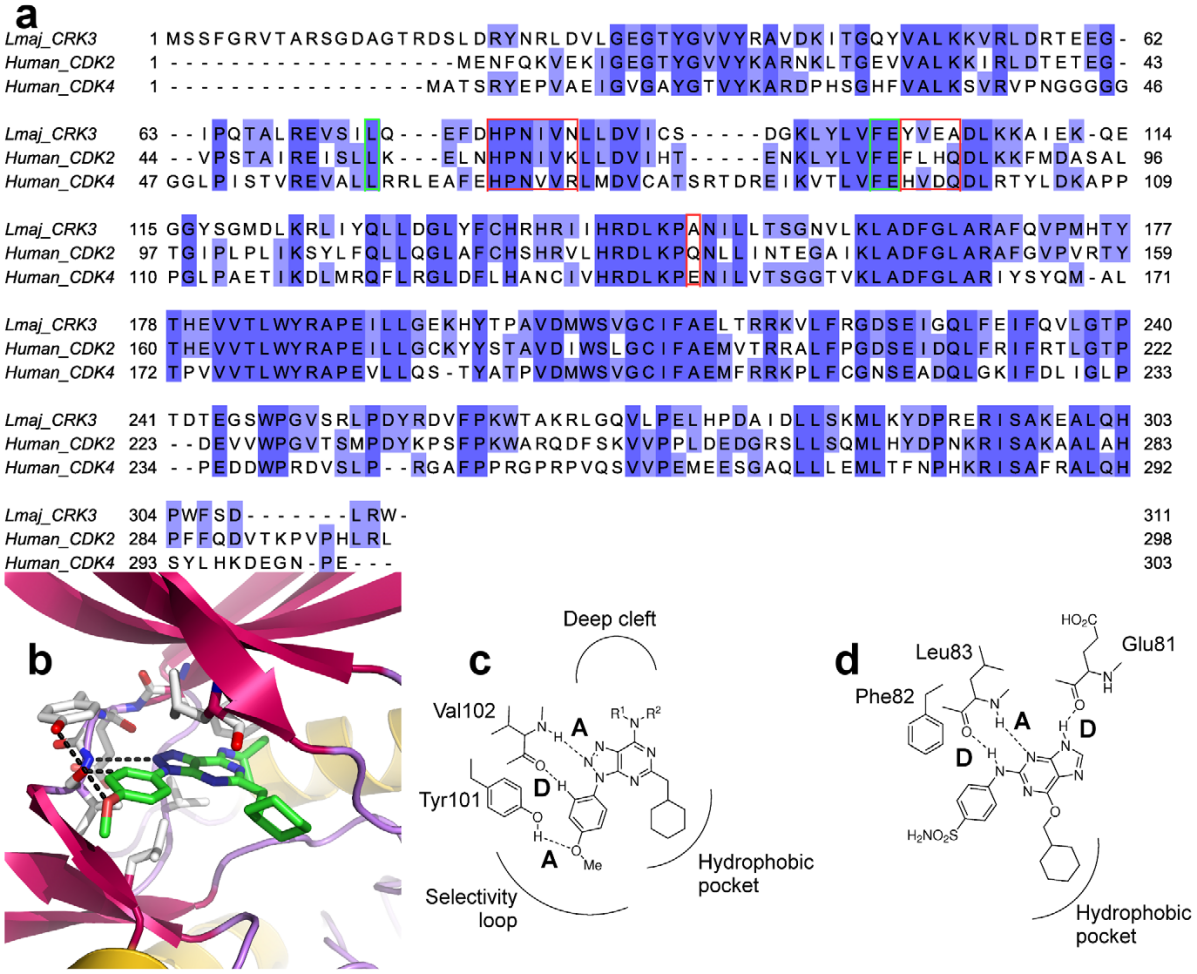
Predicted binding of azapurine pharmacophore and model of CRK3 active site. a) Sequence alignment of *Lmaj*CRK3, *Human* CDK2 and *Human* CDK4 showing the percentage identity in shades of blue. The active site regions are boxed in green with key differences boxed in red. b) A model of *Lm* CRK3 with an azapurine derivative (compound 11) docked in to the ATP site to show the predicted binding mode. c) Schematic overview of the predicted binding mode of azapurine derivatives with *Lm* CRK3 detailing the A-D-A motif not possible in CDK2 due to the Tyr101 - Phe82 difference. d) CDK2 binding mode with NU6102 [39] showing the D-A-D motif.

The 12 azapurines were screened against a panel of 10 mammalian protein kinases (Cdk1:CycB, Cdk4:CycD1, Cdk7:CycH, Cdk9:CycT1, GSK-3β, Aurora A, Plk1, Ftl3, Abl and Akt/PKB) to determine their selectivity. The 12 compounds were inactive (at 50 µM) against 10 of the 11 protein kinases tested. The one exception was Cdk4:CycD1, where all the azapurines showed inhibitory activity at <30 µM (Table 2). In the absence of the structure of CRK3:CYC6, a model of the active site of *L. major* CRK3 based on the human CDK2 structure provides a possible explanation for the specificity of the binding of the azapurines. The model was built as described in the methods section. The binding mode of kinase inhibitors has been shown to be via a hydrogen bond donor-acceptor-donor (D-A-D) motif that interacts with the backbone residues of CDK2, Leu83 and Glu81, (see Figure 2d for an example). Interestingly, the azapurine compounds have no obvious H-bond donating atom and therefore binding to the ATP pocket must be driven by hydrophobic interactions and accepting H-bonding atoms from the protein. The azapurine compounds were modelled into the CRK3 ATP site by keeping the hydrophobic interactions of the cyclohexylmethyl moiety (an area of conservation between CDK2 and CRK3) and placing N^7^ and N^8^ of the triazole moiety within the limits of H-bond accepting to the backbone of Val102 (Leu83 in CDK2). The result showed that the O atom of the methoxybenzene group is situated in a position whereby it is able to H-bond with Tyr101. A third weak H-bonding interaction is predicted between an aromatic H-atom and the backbone carbonyl of Val102. Three H-bonding interactions are evident between CRK3 and the azapurine inhibitors (Figure 2b and c), but the motif is changed to A-D-A. This A-D-A binding motif is not possible in CDK2 where Tyr101 is replaced by Phe82, which is unable to donate an H-bonding atom. In CDK4, the tyrosine residue is replaced by histidine (see Figure 2a for an alignment), which would still be able to facilitate the A-D-A binding motif; therefore, the model also explains why the azapurine compounds exhibit a lesser selectivity for CRK3 over CDK4.

To further validate the results shown in Table 2, four of the 12 azapurine compounds were re-synthesised (3, 5, 6, and 9, Table 2, Supporting Information Text S1). When screened against *Leishmania* CRK3:CYC6, the IC_50_ values returned were of 24.2 µM, 4.2 µM, 4.4 µM and 37.9 µM respectively. Compared to the original screen, compounds 5 and 6 gave comparable IC_50_ values, whilst compounds 3 and 9 exhibited a decrease in potency.

**Figure 3:**
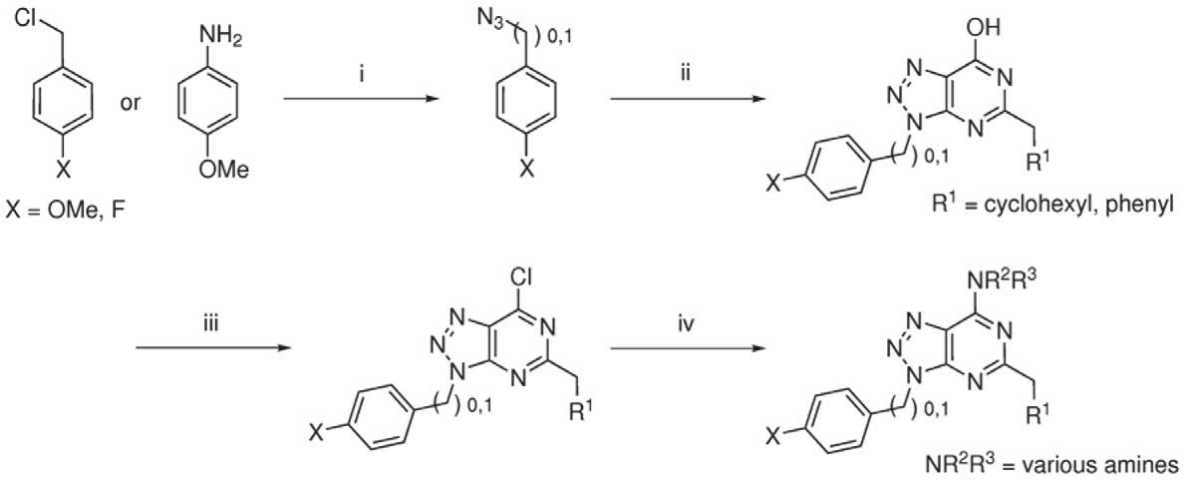
Route of synthesis of the Azapurine derivatives. (i) NaN_3_, acetonitrile, 100°C, 90 mins; or H_2_SO_4_, NaNO_2_, H_2_O, 5°C, 10 mins, then NaN_3_, hexane 5°C – RT, 2 hrs. (ii) NaOEt, ethanol, cyanoacetamide, ethyl cyclohexylacetate (or ethyl phenylacetate), 110°C, overnight. (iii) POCl_3_, microwave, 130°C, 10 mins. (iv) HNR^2^R^3^, Et_3_N, dichloromethane, microwave, 110°C, 10 mins.

In order to extend the series of compounds shown in Table 2, twenty three azapurine derivatives were synthesised (Figure 3 and Table S1 in Supporting Information Text S1) and assayed for *Leishmania* CRK3:CYC6 inhibitory activity (Table 3). Four compounds, 13, 17, 27, and 33 returned IC_50_ values <50 µM, with 13 the most active against the complex at 15.9 µM (Table 3).

**Table 3:** Azapurine derivatives.

Compound	Scaffold	CRK3:CYC6IC_50_ (µM)	IC_50_ against WT promastigote *L. major* (µM)	IC_50_ against WT amastigote *L. major* (µM)
13	A	15.9	>50	ND
14	A	>50	ND	ND
15	A	>50	ND	ND
16	A	>50	ND	ND
17	A	39.1	7.4	5–15
18	A	>50	ND	ND
19	A	>50	5–50	ND
20	A	>50	ND	ND
21	A	>50	ND	ND
22	B	>50	ND	ND
23	B	>50	ND	ND
24	A	>50	ND	ND
25	B	>50	ND	ND
26	B^1^	>50	ND	ND
27	A	30.3	28.7	15–30
28	A	>50	8.3	5–15
29	B	>100	38.7	Activity at 25 µM
30	B	>100	3.8	Activity at 10 µM
31	B	>20	40	Activity at 25 µM
32	B	>100	>50	>25
33	A	47.6	>50	>50
34	A	>50	8.3	5–15
35	A	>50	>50	>50

1 Compound 26 contains a 4-bromo substitution on the benzyl moiety of scaffold B (See Table S3 in Supporting Information Text S1 for compound structures). ND, not determined.

### Testing azapurine compounds against *L. major*

Eight of the most active azapurines were screened against wild type *L. major*, both promastigote and amastigote life cycle stages, in cell based assays. This highlighted two compounds with activity towards the parasite. Compound 5, which had activity towards the promastigote life cycle stage of *Leishmania* returning an IC_50_ value of 8.6 µM, with no activity towards the amastigote life cycle stage (Table 2). Conversely, compound 2 did not have activity towards promastigote WT *L. major*, but did exhibit some activity towards the amastigote life cycle stage returning an IC_50_ value of 38.4 µM (Table 2).

Of the azapurine derivatives synthesised, eight compounds showed a range of activity towards promastigote *L. major*: 17, 19, 27, 28, 29, 30, 31, and 34. The most potent compound against *L. major* promastigotes was compound 30 with an IC_50_ value of 3.8 µM (Table 3). The compounds with the most activity against intra-macrophage amastigotes were 17 and 28 with IC_50_ values of 5–15 µM (Table 3).

### BioFocus SFK48 library HTS screen of *Leishmania* CRK3:CYC6

In order to identify compounds with greater activity towards *Leishmania* CRK3:CYC6 and WT *L. major*, a second HTS was carried out with a kinase focussed chemical library, SFK48 comprising 528 compounds, from BioFocus. The library was screened against *Leishmania* CRK3:CYC6 at a primary concentration of 20 µM and counter screened against human CDK2:CycA. Thirty six compounds were identified which inhibited *Leishmania* CRK3:CYC6, a hit rate of 6.6% for this library. Of the 36 compounds, 13 were selective for *Leishmania* CRK3:CYC6 versus human CDK2:CycA and were thiazole compounds. The thiazole pharmacophore is shown in Table 4. Further quantities of 11 compounds were repurchased from BioFocus, seven from the 13 showing selectivity towards *Leishmania* CRK3:CYC6 (table 4, compounds 36–42), and four control compounds, two of which were active towards both CRK3:CYC6 and CDK2:CycA (compounds 43 and 44), and two that were inactive towards both CRK3:CYC6 and CDK2:CycA (compounds 45 and 46) in the primary screen. The seven compounds showing selectivity were rescreened against CRK3:CYC6 to confirm their activity, displaying IC_50_ values ranging from 3.5–10 µM (Table 4). They were also re-screened against CDK2:CycA and all had IC_50_ values above 20 µM (Table 4).

**Table 4:** BioFocus SFK48 HTS hits.

Compound	CRK3:CYC6IC_50_ (µM)	CDK2:CYCAIC_50_ (µM)	IC_50_ against WT promastigote *L. major* (µM)
36	3.5	>20	26.8
37	4.8	>20	27.5
38	5.0	>20	3.3
39	7.5	>20	3.8
40	7.8	>20	>50
41	9.0	>20	26.1
42	10.0	>20	6.9
43	ND	ND	12.5
44	ND	ND	14.4
45	ND	ND	6.8
46	ND	ND	7.8

ND, not determined. (See Table S3 in Supporting Information Text S1 for compound structures).

### Testing BioFocus SFK48 compounds against *L. major*

All 11 compounds were screened against promastigote WT *L. major* to determine their biological activity. Ten of the compounds exhibited activity towards WT *L. major* with only one returning an IC_50_ value >50 µM (Table 4, compound 40). The most potent compound was compound 38 with an IC_50_ value of 3.3 µM. Although inactive against CRK3:CYC6, compounds 45 and 46 were active against WT promastigote *L. major* with IC_50_ values of 6.8 µM and 7.8 µM, respectively. This is most probably due to the compounds hitting another target in *Leishmania*, possibly another kinase. 10 µM compounds 36–39 and 41–46 were found to be toxic to murine macrophages, so activity against amastigotes could not be assessed.

## Discussion

The work presented here describes the preparation of an active leishmanial CDK complex, the development of an assay suitable for high-throughput screening and the results of two chemical library screens, including the identification of a new class of CRK3 inhibitor, the azapurines. Previous small scale chemical library screens against *L. mexicana* CRK3 used active complex purified from transgenic parasites, expressing a his-tagged version of CRK3 [38]. Although this preparation was useful in the preliminary validation of CRK3 as a potential drug target in *Leishmania*, it was not suitable for high throughput screening. The complex was only stable for short periods of time necessitating repeated purification, introducing the possible complication of batch to batch variation. Moreover, CRK3 purified from transgenic *Leishmania* was a heterogeneous mix, likely to contain both monomer and complex, in unknown proportions. In addition, CRK3 is known to bind at least two cyclins (CYCA and CYC6) and it may also be present in more than one phosphorylation state (which would be predicted to both activate and inactivate the complex). Since we could not characterise or control the relative proportions of each component in this heterogeneous mixture, we sought alternative ways in which to produce active CRK3 complex. Since *L. mexicana* CRK3 has been shown to function predominantly in the G2/M phase of the cell cycle [20] and CYC6 in *T. brucei* was the mitotic cyclin partner for TbCRK3, we focused on the leishmanial homologue of TbCYC6 [4].

Initially, CRK3 and CYC6 were expressed (his-tagged) and purified separately in bacteria. These were then combined *in vitro* and found to form an active complex. Once it was established that CYC6 could bind and activate CRK3, we pursued the co-expression of CRK3his and CYC6his together in bacteria, in an attempt to overcome the low expression of CYC6 when expressed on its own (Figure 1A, lane 2). This approach was successful, but because both subunits were his-tagged and CRK3 was expressed at higher concentrations, the resultant purified preparation contained an excess of CRK3 (Figure 1B) and therefore consisted of a mixture of complex and monomer. The complex was separated from monomer by gel filtration chromatography and was used to perform the library screens. Subsequently, we co-expressed his-tagged CYC6 and non-tagged CRK3, purifying initially by Ni-chelate column chromatography, such that CYC6his, and CRK3 in complex with the cyclin, would be retained on the column whilst monomer CRK3 would be eluted. The resultant preparation is a 1∶1 molar ratio of CRK3 and CYC6 (Figure 1C), was extremely stable on storage and is being used to attempt to crystallise the complex. Unfortunately, despite assessing a wide range of conditions, no CRK3:CYC6 crystals have been obtained to date.

Once a defined and reproducible source of active CRK3 had been established, assays were developed, both radiometric and fluorescence polarisation. The IMAP fluorescence polarisation assay was chosen to screen the chemical libraries because it required 6 times less enzyme per reaction than the radiometric assay and because a fluorescence based platform is more suitable for an HTS campaign. A number of peptide substrates were phosphorylated by CRK3 but all of them complied with the consensus phosphorylation pattern for CDKs: x_−1_(S/T_0_)P_+1_x_+2_(K/R_+3_) [36], indicating that the recognition and phosphor-transfer mechanism is conserved in the leishmanial CDK.

Both chemical library screens (Lexicon and BioFocus SFK48) yielded inhibitors of CRK3. CRK3 was screened against the 25,000 compound Lexicon library at a single 10 µM concentration and counter screened against CDK2:CycA. Only specific inhibitors of *Leishmania* CRK3 were sought, as a previous small scale screen of anti-mitotic compounds had identified many CRK3 inhibitors, but none that had specificity in comparison with mammalian CDK homologues [38]. 37 compounds were confirmed as inhibitors of CRK3. Twelve of the most potent CRK3 inhibitors were azapurine compounds. Comparison of these active azapurine compounds with other azapurine compounds in the library which did not inhibit CRK3 revealed that the active compounds all had a methoxybenzene group at the 9-position and a cyclohexylmethyl group at position 2 (Table 2). Interestingly, during counter-screening, these compounds were also found to be inactive against 10 out of 11 mammalian kinase enzymes tested, with the exception of CDK4/CycD1. Modelling of the azapurine compounds into the active site of CRK3 revealed a possible explanation for this selective inhibition of the parasite kinase. Instead of the normal donor-acceptor-donor binding motif used by other kinase inhibitors, the azapurines are predicted to bind to CRK3 using an acceptor-donor-acceptor (A-D-A) motif, which is not possible in the mammalian protein kinases tested (apart from CDK4/CycD1), see Figure 2. Moreover, this binding motif is consistent with the requirement for a methoxybenzene group at position 9, which is involved in hydrogen bonding to Tyr101, and with the requirement for the non-polar cyclohexylmethyl group at position 2, which can then form hydrophobic interactions with the hydrophobic pocket (Figure 2b and 2c).

Although all the azapurine CRK3 inhibitors also inhibited CDK4/CycD1, the relative potencies toward the 2 enzymes varied between compounds. For instance, compound 2 was equally active against both CRK3 and CDK4/CycD1 but compound 12 was more potent against the mammalian CDK (Table 2). Since all the Lexicon azapurines in Table 2 have a methoxybenzene at position 9 and cyclohexylmethyl at position 2, they differ only in their substituent group at position 6 (Table 2) implying that the small differences in potency of these compounds towards CRK3 and CDK4/CycD1 must be due to differences in the “deep cleft” of these two kinases. This opens up the possibility of exploiting these differences to design an azapurine inhibitor with more favourable parasite selectivity.

A number of other azapurine derivatives were synthesised in order to explore further the azapurine scaffold and test the binding hypothesis. None of these derivatives were more potent than the original hits from the chemical library screen. They did, however, provide some useful structure activity data. In compound 33 (Table 3), the methoxybenzene group at position 9 was successfully replaced with a fluorobenzene group, in which the electronegative fluorine can act as a hydrogen bond acceptor in place of the oxygen of the methoxybenzene, thus maintaining the A-D-A binding motif. Introduction of an additional methyl group between the azapurine core structure and the methoxybenzene ring resulted in loss of the CRK3-inhibitory activity – compare compounds 13 with 14 and 20 with 27. This can be explained with reference to the A-D-A binding motif, in that the introduction of an additional methyl group, would shift the position of the oxygen atom of the methoxybenzene group such that it could no longer act as an efficient hydrogen bond acceptor from the hydroxyl group of Tyr101 (Figure 2b an 2c). Replacement of the cyclohexylmethyl group at position 2 with the aromatic methylbenzene ring resulted in a dramatic decrease in the CRK3 inhibitory activity – compare compound 31 in Table 3 (IC_50_>20 µM), with compound 6, Table 2 (IC_50_ = 6.9 µM). Although methylbenzene should also be capable of making hydrophobic interactions with the hydrophobic pocket, it would have a considerably different 3D-shape from the cycloalkane ring. A benzene ring is planar in structure, whilst the cyclohexyl ring normally adopts a contorted, energetically-favourable “chair” conformation. Perhaps the shape of the cyclohexyl ring is more “complimentary” to the shape of the hydrophobic pocket and thus is more favourable for interaction with CRK3.

The azapurine compounds were also tested against the parasite in culture; both insect-stage promastigotes and intra-macrophage amastigotes. Only one of the Lexicon azapurine compounds had any activity against the parasite *in vitro* (compound 5, IC_50_ versus promastigotes = 8.6 µM, Table 2). Of the azapurine derivatives depicted in Table 3, few inhibited parasite growth in culture (17, 27, 28, 29 and 30). Some of these compounds did not inhibit CRK3/CYC6 *in vitro*, indicating that the drug target in the parasite was unlikely to be CRK3 (Table 3, compounds 28, 29 and 20). Despite being relatively selective CRK3 inhibitors, none of the azapurine compounds displayed potent anti-parasite activity. The IC_50_ values for the compounds were in the micromolar range; perhaps they were not potent enough CRK3 inhibitors to be able to have an effect at the whole cell level. Or they may not have been able to achieve sufficiently high intracellular concentration to have an inhibitory effect on CRK3 *in vivo*. Further modification of the azapurine scaffold may yet achieve inhibitors with the correct profile of CRK3 selectivity, cell permeability and anti-parasite activity.

The screening of the second chemical library, BioFocus SFK48, yielded a better “hit rate” with 6.6% of compounds being identified as CRK3 inhibitors. This is perhaps unsurprising since this was a kinase-focused library. Of the original 36 hits, 13 compounds were identified that selectively inhibited CRK3 more than human CDK2; these compounds all contained the thiazole scaffold (Table 4). A number of thiazole compounds were tested against promastigote *L. major* and most were found to have moderate anti-parasite activity. However, there was no correlation between the activity against the parasite and activity against the purified CRK3:CYC6 enzyme complex. This might be due to differing cell permeability or because the observed effects are not due to CRK3 inhibition alone. Indeed, two compounds that did not inhibit CRK3 could inhibit parasite replication *in vitro*. Clearly, their effects are not due to inhibition of CRK3 and are most likely due to the inhibition of another protein kinase in the parasite. More work needs to be done to establish whether the growth inhibitory effects of the thiazole CRK3 inhibitors is due to inhibition of CRK3 *in vivo*, either partially or wholly.

In this study, CRK3:CYC6 cyclin-dependent kinase selective inhibitors were identified, yet poor correlation was observed between potency against the target and anti-parasite activity. Optimisation of the two series of compounds will be required to increase potency of the compounds against CRK3:CYC6, so that an assessment can be made of the potential of the azapurines and thiazoles to be developed into lead compounds for anti-leishmanial drug development activities. It remains an open question whether selectivity for the parasite target should be a priority in selection protocols for HTS screening programs, or whether potent inhibitors should be first identified and then selectivity sought in subsequent chemistry optimisation.

## Supporting Information

Text S1 Supporting information S1
(0.35 MB DOCX)
