# Supplementary material for: High Throughput Screens Yield Small Molecule Inhibitors of Leishmania CRK3:CYC6 Cyclin-Dependent Kinase
Source: PLoS Negl Trop Dis. 2011 Apr 5;5(4):e1033. doi: 10.1371/journal.pntd.0001033 (PMC3071374; doi:10.1371/journal.pntd.0001033)
Supplement: Text S1 — Supporting information S1 (0.35 MB DOCX) [file pntd.0001033.s001.docx]

**SUPPORTING INFORMATION S1**

**Figure S1 – Linearity of the *Leishmania* CRK3:CYC6 radiometric assay**. The linearity of the assay developed for the *Leishmania* CRK3:CYC6 protein kinase complex was determined over 60 minutes. 7.5ng of *Leishmania* CRK3:CYC6, 0.4mg ml^-1^ (3.6μM) histone H1 substrate and 100μM [γ-^32^P]-Mg-ATP were incubated at 30C. The assay was stopped at various time points by the addition of orthophosphoric acid. The reaction was spotted onto a P81 filter plate and following washing with orthophosphoric acid, was counted for the incorporation of radioactivity. Graphical analysis was carried out using XLfit 4.0 software from IDBS (www.idbs.com).

**Figure S2 – CRK3:CYC6 IMAP assay development and validation – enzyme titration.** Using the generic sequence peptide as the substrate, a two-fold enzyme titration was carried out. This identified that 1.25ng of CRK3:CYC6 could be used in the IMAP high throughput screen format. Graphical analysis was carried out using XLfit 4.0 software from IDBS ([www.idbs.com](http://www.idbs.com)).

**Synthesis of novel azapurine ligands**

**General methods**

Commercially available reagents were used as received without purification. Analytical thin layer chromatography was performed using Mackerey-Nagel plastic backed plates coated with 0.2 mm thick silica gel 60F_254_. The plates were visualised by UV light (254 nm) and potassium permanganate dip. Flash column chromatography was conducted with Merck silica gel 60H (40-60 μm, 230-400 mesh). A CEM Discover microwave was used for the microwave heated steps (300W max. power input). Nominal mass spectra were recorded on an Agilent 1100 series spectrometer using positive electrospray ionisation (ESI). ^1^H and ^13^C NMR spectra were recorded on a Bruker DPX 400 MHz or 300 MHz spectrometer. Chemical shifts (δ) are reported in parts per million (ppm) and coupling constants (*J*) in Hz. Residual protic solvent C*H*Cl_3_ (δ_H_ 7.26) or DMSO (δ_H_ 2.50) was used as the internal standard in ^1^H NMR spectra. The following abbreviations were used to define the multiplicities: s, singlet; d, doublet; t, triplet; q, quartet; m, multiplet; br, broad.

Azapurine synthesis


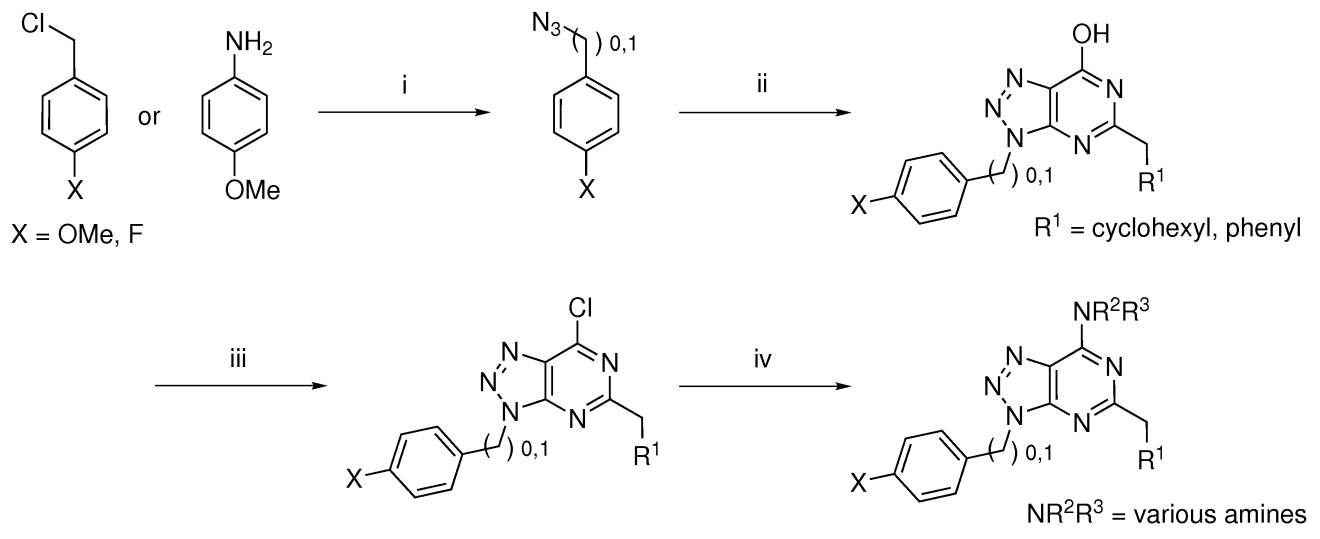


Figure S3. The synthesis of the azapurine core structures (13, 14, 22-26) was based on that published by G. Biagi *et al.*^[[1]](#footnote-1)^ (i) NaN_3_, acetonitrile, 100°C, 90 mins; or H_2_SO_4_, NaNO­­_2_, H­_2_O, 5°C, 10 mins, then NaN­_3_, hexane 5°C – RT, 2 hrs. (ii) NaOEt, ethanol, cyanoacetamide, ethyl cyclohexylacetate (or ethyl phenylacetate), 110°C, overnight. (iii) POCl_3_, microwave, 130°C, 10 mins. (iv) HNR^2^R­^3^, Et­_3_N, dichloromethane, microwave, 110°C, 10 mins.

**General procedure to prepare 2,9-disubstituted-6-amino-8-azapurines (15-21, 27-35).**

2,9-disubstituted-6-hydroxy-8-azapurine (0.3 mmol) was suspended in phosphorous oxychloride (1.0 ml) in a suitable microwave reaction vessel and heated to 130°C for 10 minutes under microwave irradiation. The reaction mixture was allowed to cool, and excess phosphorous oxychloride was removed *in vacuo*. The residue was dissolved in dichloromethane (10 ml) and washed with ice cold water (5 ml), and the organic layer separated and dried (Na_2_SO_4_). The drying agent was filtered off, and the dichloromethane removed *in vacuo* to give a solid that was used crude in the next step. 2,9-disubstituted-6-chloro-8-azapurine was dissolved in dichloromethane (2 ml) and the required amine (1.5 eq.) and triethylamine (2.0 eq.) were added, and the reaction mixture heated to 110°C for 10 minutes under microwave irradiation. The reaction mixture was allowed to cool, followed by purification by flash chromatography.

Table S1. Analytical data for novel azapurine derivatives.

| 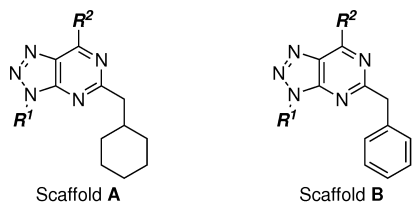 | | | | | | |
| --- | --- | --- | --- | --- | --- | --- |
| **Compound** | **Scaffold** | ***R^1^*** | ***R^2^*** | **Yield** | **MS** | **_­_^1^H NMR** |
| **13** | A | 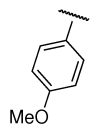 | 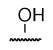 | 59% | 340.3 | 7.86 (d, *J* = 8.9 Hz, 2H), 7.16 (d, *J* = 8.9 Hz, 2H), 3.82 (s, 3H), 2.49 (d, *J* = 16.6 Hz, 2H), 1.92 – 1.69 (m, 1H), 1.73 – 1.43 (m, 5H), 1.33 – 0.75 (m, 5H).^a, i^ |
| **14** | A | 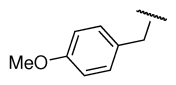 | 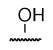 | 27% | 354.1 | 12.38 (s, 1H), 7.45 (d, *J* = 8.0, 2H), 6.88 (d, *J* = 8.0, 2H), 5.67 (s, 2H), 3.71 (s, 3H), 2.76 (d, *J* = 7.0, 2H), 1.94-2.05 (m, 1H), 1.65-1.80 (m, 5H), 1.06-1.36 (m, 5H).^b, ii^ |
| **15** | A | 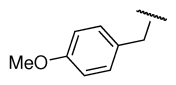 | *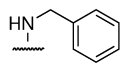* | 47% | 443.2 | 7.49 – 7.16 (m, 7H), 6.84 (d, *J* = 7.9 Hz, 2H), 5.63 (s, 2H), 5.36 (s, br, 1H), 4.87 (d, *J* = 5.2 Hz, 2H), 3.77 (s, 3H), 2.74 (d, *J* = 7.1 Hz, 2H), 2.10 – 1.85 (m, 1H), 1.80 – 1.48 (m, 5H), 1.37 – 0.92 (m, Hz, 5H). ^b, i^ |
| **16** | A | 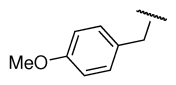 | 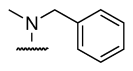 | 95% | 457.9 | 7.46 (d, *J* = 7.3 Hz, 2H), 7.39 – 7.21 (m, 5H), 6.85 (d, *J* = 7.3 Hz, 2H), 5.64 (s, 2H), 5.09 (s, 2H), 3.78 (s, 3H), 3.76 (s, 3H), 2.73 (d, *J* = 7.0 Hz, 2H), 1.91 – 2.08 (m, 1H), 1.58 – 1.80 (m, 5H), 1.45 – 0.88 (m, 5H). ^b, i^ |
| **17** | A | 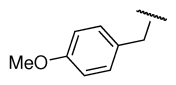 | 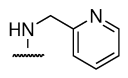 | 74% | 445.1 | 8.58 (ddd, *J* = 4.9, 1.5, 0.8 Hz, 1H), 7.65 (t, *J* = 7.5 Hz, 1H), 7.47 – 7.31 (m, 3H), 7.24 – 7.14 (m, 1H), 6.82 (d, *J* = 8.5 Hz, 2H), 5.65 (s, 2H), 4.96 (d, *J* = 4.9 Hz, 2H), 3.76 (s, 3H), 2.72 (d, *J* = 7.2 Hz, 2H), 2.05 – 1.87 (m, 1H), 1.77 – 1.50 (m, 5H), 1.34 – 0.88 (m, 5H). ^b, ii^ |
| **18** | A | 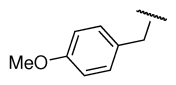 | 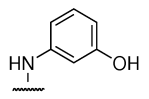 | 71% | 445.1 | 8.28 (s, br, 1H), 7.57 – 7.50 (m, 1H), 7.49 – 7.35 (m, 4H), 7.22 (t, *J* = 8.1 Hz, 1H), 6.84 (d, *J* = 8.1, 2H), 6.65 (ddd, *J* = 8.1, 2.4, 0.7 Hz, 1H), 5.69 (s, 2H), 3.76 (s, 3H), 2.81 (d, *J* = 7.2 Hz, 2H), 2.13 – 1.96 (m, 1H), 1.78 – 1.45 (m, 5H), 1.30 – 0.90 (m, 5H). ^b, ii^ |
| **19** | A | 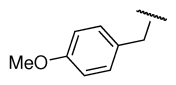 | 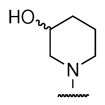 | 68% | 437.1 | 7.41 (d, *J* = 8.7 Hz, 2H), 6.82 (d, *J* = 8.7 Hz, 2H), 5.63 (s, 2H), 4.76 – 4.18 (m, br, 3H), 4.15 – 3.82 (m, br, 2H), 3.75 (s, 3H), 2.67 (d, *J* = 7.1 Hz, 2H), 2.32 (s, br, 1H), 2.06 – 1.83 (m, 3H), 1.83 – 1.48 (m, 7H), 1.35 – 1.09 (m, 3H), 1.11 – 0.93 (m, 2H). ^b, ii^ |
| **20** | A | 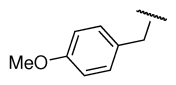 | 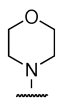 | 66% | 423.2 | 7.44 (d, *J* = 8.8 Hz, 1H), 6.83 (d, *J* = 8.8 Hz, 2H), 5.73 (s, 2H), 4.83 – 4.57 (m, 2H), 4.24 – 4.00 (m, 2H), 3.93 – 3.81 (m, 4H), 3.76 (s, 3H), 2.78 (d, *J* = 7.2 Hz, 2H), 2.01 – 1.85 (m, 1H), 1.76 – 1.59 (m, 5H), 1.31 – 0.98 (m, 5H). ^b, ii^ |
| **21** | A | 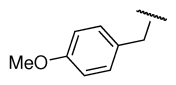 | 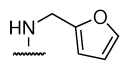 | 72% | 433.2 | 7.58 – 7.32 (m, 3H), 6.83 (d, *J* = 8.3 Hz, 2H), 6.48 (s, br 1H), 6.31 (s, br, 2H), 5.65 (s, 2H), 4.86 (d, *J* = 3.9 Hz, 2H), 3.76 (s, 3H), 2.75 (d, *J* = 6.5 Hz, 2H), 2.02 – 1.55 (m, 6H), 1.34 – 0.93 (m, 5H). ^b, i^ |
| **22** | B | 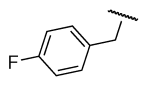 | 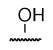 | 68% | 337.5 | 12.84 (s, 1H), 7.43 – 7.22 (m, 7H), 7.21 – 7.10 (m, 2H), 5.67 (s, 2H), 4.01 (s, 2H). ^a, ii^ |
| **23** | B | 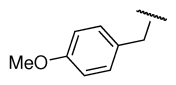 | 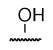 | 57% | 348.1 | 12.83 (s, 1H), 7.47 – 7.14 (m, 7H), 6.88 (d, *J* = 8.5 Hz, 2H), 5.60 (s, 2H), 4.02 (s, 2H), 3.73 (s, 3H). ^a, ii^ |
| **24** | A | 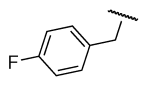 | 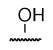 | 52% | 342.1 | 12.55 (s, 1H), 7.52 – 7.27 (m, 2H), 7.17 (t, *J* = 8.7 Hz, 2H), 5.70 (s, 2H), 2.52 (d, *J* = 7.0 Hz, 2H), 1.93 – 1.71 (m, 1H), 1.71 – 1.44 (m, 5H), 1.30 – 0.77 (m, 5H). ^a, i^ |
| **25** | B | 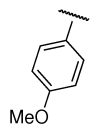 | 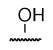 | 54% | 334.2 | 12.97 (s, 1H), 7.87 (d, *J* = 8.4 Hz, 2H), 7.46 – 7.19 (m, 5H), 7.15 (d, *J* = 8.4 Hz, 2H), 4.02 (s, 2H), 3.83 (s, 3H). ^a, ii^ |
| **26** | B^1^ | 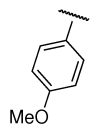 | 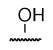 | 45% | - | 7.88 (d, *J* = 8.5 Hz, 2H), 7.52 (d, *J* = 7.8 Hz, 2H), 7.33 (d, *J* = 7.8 Hz, 2H), 7.17 (d, *J* = 8.5 Hz, 2H), 4.01 (s, 2H), 3.85 (s, 3H). ^a, ii^ |
| **27** | A | 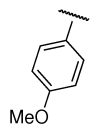 | 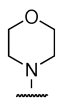 | 78% | 410.1 | 8.09 (d, *J* = 9.1 Hz, 2H), 7.08 (d, *J* = 9.1 Hz, 2H), 4.90 – 4.49 (m, 2H), 4.32 – 3.98 (m, 2H), 3.99 – 3.77 (m, br, 7H), 2.70 (d, *J* = 7.2 Hz, 2H), 2.07 – 1.83 (m, 1H), 1.81 – 1.51 (m, 5H), 1.38 – 0.93 (m, 5H). ^b, ii^ |
| **28** | A | 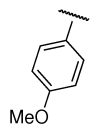 | 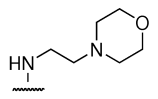 | 79% | 453.0 | 8.03 (d, *J* = 9.0 Hz, 2H), 7.00 (d, *J* = 9.0 Hz, 2H), 6.76 (t, br, *J* = 4.6 Hz, 1H), 3.81 (s, 3H), 3.76 – 3.64 (m, 6H), 2.79 – 2.58 (m, 4H), 2.56 – 2.35 (m, 4H), 1.98 – 1.82 (m, 1H), 1.77 – 1.47 (m, 5H), 1.32 – 0.81 (m, 5H). ^b, ii^ |
| **29** | B | 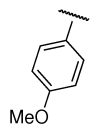 | 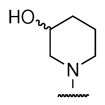 | 70% | 417.0 | 7.96 (d, *J* = 8.6 Hz, 2H), 7.41 – 7.32 (m, 2H), 7.28 (t, *J* = 7.4 Hz, 2H), 7.23 – 7.13 (m, 3H), 5.08 (d, *J* = 4.4 Hz, 1H), 4.03 (s, 2H), 3.85 (s, 3H), 2.01 – 1.76 (m, br, 2H), 1.62 – 1.39 (m, br, 2H), [5.16 – 5.09, 5.02 – 4.94, 4.72 – 4.65, 4.30 – 4.23, 4.01 – 3.94, 3.81 – 3.51, 3.22 – 3.14 (m, br, rotamers, 5H)]. ^a, ii^ |
| **30** | B | 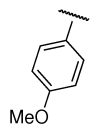 | 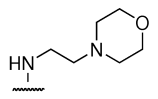 | 76% | 446.0 | 8.87 (t, br, *J* = 5.6 Hz, 1H), 8.00 (d, *J* = 8.8 Hz, 2H), 7.41 – 7.12 (m, 7H), 4.05 (s, 2H), 3.85 (s, 3H), 3.65 (dd, *J* = 12.4, 6.2 Hz, 2H), 3.54 – 3.13 (m, 6H), 2.53-2.48 (m, 2H), 2.40 – 2.33 (m, 2H). ^a, ii^ |
| **31** | B | 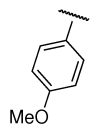 | 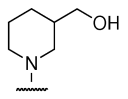 | 80% | 431.0 | 7.96 (d, *J* = 7.7 Hz, 2H), 7.44 – 7.12 (m, 7H), 5.53 (dd, *J* = 47.9, 12.3 Hz, 1H), 4.94 (dd, *J* = 53.6, 12.3 Hz, 1H), 4.69 (t, *J* = 5.2 Hz, 1H), 4.04 (s, 2H), 3.85 (s, 3H), 3.41 – 3.28 (m, 4H), 1.96 – 1.26 (m, 5H). ^a, ii^ |
| **32** | B | 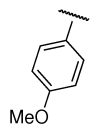 | 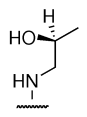 | 76% | 391.2 | 8.86 (s, 1H), 8.00 (d, *J* = 8.6 Hz, 2H), 7.46 – 7.10 (m, 7H), 4.84 (s, 1H), 4.05 (s, 2H), 3.85 (s, 3H), 3.57 – 3.30 (m, 3H), 1.07 (d, *J* = 6.0 Hz, 2H). ^a, ii^ |
| **33** | A | 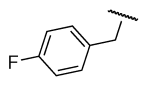 | 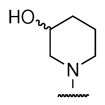 | 71% | 425.5 | 7.54 – 7.24 (m, 2H), 7.16 (t, *J* = 8.5 Hz, 2H), 5.72 (s, 2H), 3.78-3.04 (m, 5H), 2.59 (d, *J* = 6.9 Hz, 2H), 2.05 – 0.70 (m, br, 15H). ^a, i^ |
| **34** | A | 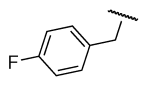 | 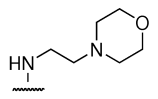 | 84% | 354.3 | 8.77 (t, *J* = 5.6 Hz, 1H), 7.48 (dd, *J* = 8.6, 5.6 Hz, 2H), 7.24 (t, *J* = 8.6 Hz, 2H), 5.79 (s, 2H), 3.73 (dd, *J* = 12.4, 6.3 Hz, 2H), 3.67 – 3.53 (m, 4H), 3.41 (s, 2H), 2.76 – 2.54 (m, 6H), 2.10 – 1.83 (m, 1H), 1.84 – 1.47 (m, 5H), 1.48 – 0.79 (m, 5H). ^a, i^ |
| **35** | A | 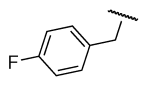 | 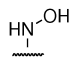 | 64% | 357.4 | 10.93 (s, 1H), 10.27 (s, 1H), 7.55 – 7.26 (m, 2H), 7.26 – 7.02 (m, 2H), 5.56 (s, 2H), 2.52 (d, *J* = 7.2 Hz, 2H), 1.83 – 1.51(m, 6H), 1.27 – 0.82 (m, 5H). ^a, i^ |
| ^1^Compound **26** contains a 4-bromo on the benzyl moiety of scaffold B. ^a^ Solvent = DMSO. ^b^ Solvent = CDCl_3_. ^i^ 300 MHz NMR, ^ii^ 400 MHz NMR. MS values refer to the observed m/z peak corresponding to the (MH)^+^ ion. | | | | | | |

Table S2. Lexicon azapurine HTS hits

| **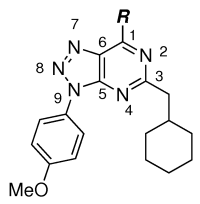** | | | | | |
| --- | --- | --- | --- | --- | --- |
| **Compound** | ***R*** | **CRK3:CYC6**  **IC_50_ (μM)** | **CDK4:CYCD1**  **IC_50_ (μM)*^a^*** | **IC_50_ against WT promastigote *L. major* (μM)** | **IC_50_ against WT amastigote *L. major* (μM)** |
| 1 | 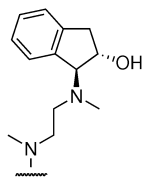 | 2.6 | 12.5 | >10 | >50 |
| 2 | 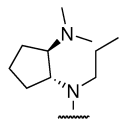 | 3.4 | 5.6 | >10 | 38.4 |
| 3 | 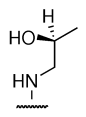 | 4.4 | 21.9 | >10 | >50 |
| 4 | 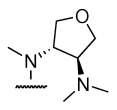 | 4.4 | 10.3 | >10 | >50 |
| 5 | 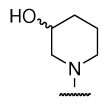 | 5.3 | 9.7 | 8.6 | >50 |
| 6 | 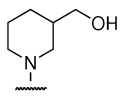 | 6.9 | 9.2 | >10 | >50 |
| 7 | 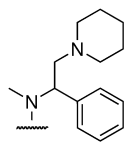 | 7.8 | 19.5 | ND | ND |
| 8 | 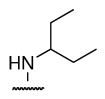 | 8.1 | 26.9 | ND | ND |
| 9 | 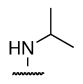 | 8.8 | 19.8 | ND | ND |
| 10 | 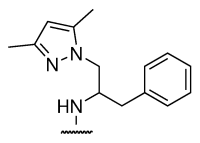 | 10.1 | 7.3 | ND | ND |
| 11 | 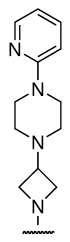 | 10.3 | 6.7 | ND | ND |
| 12 | 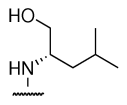 | 10.7 | 4.6 | ND | ND |
| ***^a^***Also screened against CDK1:CYCB and CDK2:CYCA; IC_50_ values for all compounds in these screens were >50 μM. | | | | | |

The compound structures and biological data are shown for the HTS hits. Compounds 7-12 displaying ND (not determined) did not have their IC_50_ values determined against *L. major* promastigotes or amastigotes due to there being insufficient compound material to do so (maximum concentration tested was 10 μM). The ± values represent the standard deviation for each of the average determinations.

Table S3 –Azapurine derivatives**.**

| 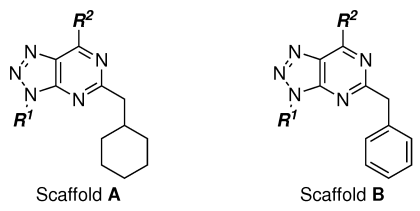 | | | | | | |
| --- | --- | --- | --- | --- | --- | --- |
| **Compound** | **Scaffold** | ***R^1^*** | ***R^2^*** | **CRK3:CYC6**  **IC_50_ (μM)** | **IC_50_ against WT promastigote *L. major* (μM)** | **IC_50_ against WT amastigote *L. major* (μM)** |
| 13 | A | 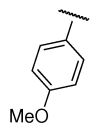 | 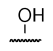 | 15.9 | >50 | ND |
| 14 | A | 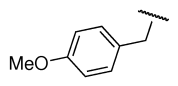 | 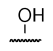 | >50 | ND | ND |
| 15 | A | 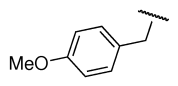 | 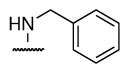 | >50 | ND | ND |
| 16 | A | 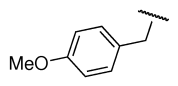 | 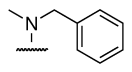 | >50 | ND | ND |
| 17 | A | 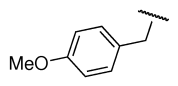 | 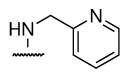 | 39.1 | 7.4 | 5-15 |
| 18 | A | 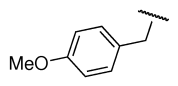 | 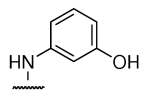 | >50 | ND | ND |
| 19 | A | 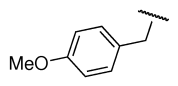 | 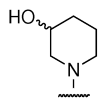 | >50 | 5-50 | ND |
| 20 | A | 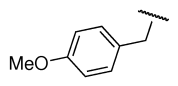 | 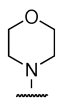 | >50 | ND | ND |
| 21 | A | 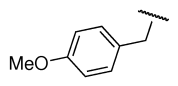 | 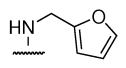 | >50 | ND | ND |
| 22 | B | 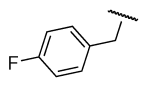 | 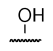 | >50 | ND | ND |
| 23 | B | 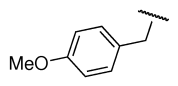 | 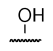 | >50 | ND | ND |
| 24 | A | 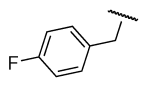 | 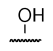 | >50 | ND | ND |
| 25 | B | 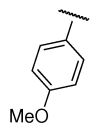 | 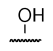 | >50 | ND | ND |
| 26 | B^1^ | 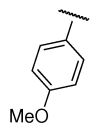 | 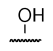 | >50 | ND | ND |
| 27 | A | 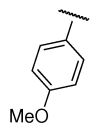 | 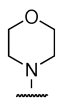 | 30.3 | 28.7 | 15-30 |
| 28 | A | 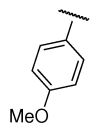 | 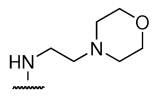 | >50 | 8.3 | 5-15 |
| 29 | B | 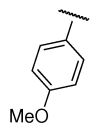 | 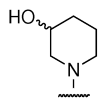 | >100 | 38.7 | Activity observed at  25 μM |
| 30 | B | 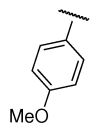 | 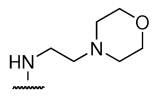 | >100 | 3.8 | Activity observed at  10 μM |
| 31 | B | 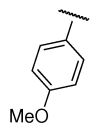 | 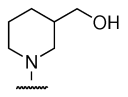 | >20 | 40 | Activity observed at  25 μM |
| 32 | B |  |  | >100 | >50 | >25 |
| 33 | A |  |  | 47.6 | >50 | >50 |
| 34 | A |  |  | >50 | 8.3 | 5-15 |
| 35 | A |  |  | >50 | >50 | >50 |
| ^1^Compound 26 contains a 4-bromo substitution on the benzyl moiety of scaffold B | | | | | | |

The compound structures and biological data are shown for the azapurine derivatives synthesised. Compounds displaying ND (not determined) did not have IC_50_ values determined against *L. major* promastigotes or amastigotes due to their lack of activity against either the CRK3:CYC6 protein kinase complex or *L. major* promastigotes, respectively.

Table S4. BioFocus SFK48 HTS hits.

|  | | | | | |
| --- | --- | --- | --- | --- | --- |
| **Compound** | ***R^1^*** | ***R^2^*** | **CRK3:CYC6**  **IC_50_ (μM)** | **CDK2:CYCA**  **IC_50_ (μM)** | **IC_50_ against WT promastigote *L. major* (μM)** |
| 36 |  |  | 3.5 | >20 | 26.8 |
| 37 |  |  | 4.8 | >20 | 27.5 |
| 38 |  |  | 5.0 | >20 | 3.3 |
| 39 |  |  | 7.5 | >20 | 3.8 |
| 40 |  |  | 7.8 | >20 | >50 |
| 41 |  |  | 9.0 | >20 | 26.1 |
| 42 |  |  | 10.0 | >20 | 6.9 |
| 43 |  |  | ND | ND | 12.5 |
| 44 |  |  | ND | ND | 14.4 |
| 45 |  |  | ND | ND | 6.8 |
| 46 |  |  | ND | ND | 7.8 |

The compound structures and biological data are shown for the SFK48 HTS hits. Compounds that were tested against *L. major* promastigotes but not the CRK3:CYC6 protein kinase complex (ND) was due to limited material. Similarly, a lack of material prevented compound testing against *L. major* amastigotes.

1. G. Biagi, A. M. Bianucci, A. Coi, B. Costa, L. Fabbrini, I. Giorgi, O. Livi, I. Micco, F. Pacchini, E. Santini, M. Leonardi, F.A.Nofal, O. L. Salernid, V. Scartoni, Bioorg. Med. Chem. 13 (2005) 4679–4693 [↑](#footnote-ref-1)
